# Diabetes effect on Quality of Life in the long-term after Limb salvage with Infrageniculate Bypasses accompanied with minor amputations

**DOI:** 10.12669/pjms.305.5226

**Published:** 2014

**Authors:** Kivanc Derya Peker, Murat Aksoy

**Affiliations:** 1 Kivanc Derya Peker, Peripheral Vascular Surgery Unit, Department of General Surgery, Medical Faculty of Istanbul, Istanbul University, Turkey.; 2 Murat Aksoy, Peripheral Vascular Surgery Unit, Department of General Surgery, Medical Faculty of Istanbul, Istanbul University, Turkey.

**Keywords:** Buerger’s Disease, Diabetes Mellitus, Peripheral Vascular Disease, Quality of Life

## Abstract

***Objective*** ***: ***To evaluate the quality of life in patients, who had their limbs salvaged with an infrageniculate bypass and minor amputation in the long term and to see if diabetics are prone to worse results.

***Methods: ***The patients with limb salvage following an infrageniculate bypass and minor amputation were asked to complete Short Form 36 at the last follow-up visit. The mean scores in diabetic and non-diabetic population were compared to each other .The mean follow-up period was 58±8 months.

***Results*** *: *Of 142 patients, 40 patients were eligible to be included in the study. 33 (82.5%) patients were male and 7 (17.5%) patients were female. The mean age at the time of intervention was 57±14 (33-83) years. The mean scores for eight domains of SF-36 evaluation ranged from 44 to 67 out of 100. There were no significant differences concerning the mean scores of any dimension between the diabetic and non-diabetic group.

***Conclusion*** **: **Despite a minor amputation, the functional outcome of limb salvage with an infrageniculate bypass is favorable and diabetes does not seem to have negative effect on the functional outcome and Quality of Life.

## INTRODUCTION

Peripheral arterial disease (PAD) is a common disease in the population. The frequency rises with advancing age and the frequency of the disease may reach up to 20% among the octogenarians.^1^ A certain percentage of patients with PAD may progress to critical limb ischemia. Although, the morbidity and mortality rates are higher in critical limb ischemia, successful lower limb revascularization is effective in preventing major amputation.^2^

Primary or secondary rates and limb salvage rates have been measures of success in vascular surgery for many years. However, these measures are for the most part physician-oriented measures and they do not necessarily reflect the outcome, which is more relevant to the patient himself/herself. Moreover, it has been well documented that surviving patients with salvaged limbs may present with impaired functional capabilities, which may necessitate significant care.^3^ Therefore, primary outcome measures that are relevant to the patient may be the functional outcome of the limb and/or quality of life (QoL). These are more patient-oriented outcome measures.

Infrainguinal bypass procedures may help to improve QoL in patients with critical ischemia. Nevertheless, these papers concern mainly the early period and not all patients in these papers suffered a minor amputation along with the intervention. There is also a conflict of the results in subgroups such as patients with diabetes and without diabetes. This article aims to evaluate the effect of crural and pedal bypasses on quality of life and assess the functional outcome of the extremity, which is salvaged with a distal bypass and minor amputation.

## METHODS

All consecutive patients, who had undergone either a crural or pedal bypass accompanied with minor amputation because of Fontaine Class IV critical limb ischemia, from 1999 to 2009 at Department of General Surgery, Istanbul Medical Faculty, Istanbul University were retrospectively evaluated. After the local ethical committee approval (Registration No.2010/224-32); patients, who were alive or did not undergo major amputation during the follow-up were included in the study. Patients, who were eligible for the study, were asked to give informed consent and complete Short Form-36 (SF-36) at the last follow-up visit. Those, who refused to do so, were excluded from the study.

The SF-36 questionnaire is a self-assessment tool for quality of life. It is a generic assessment tool and it is not disease specific. However, since peripheral arterial disease (PAD) is a multisystemic disease, it can be used for PAD. It has been validated in studies concerning patients with PAD.^4^ This instrument covers eight dimensions of health-related quality of life: physical functioning, role-physical, bodily pain, social functioning, mental health, role-emotional, vitality, and general health perceptions. The evaluation is mainly on individual basis and the scores range from 0 to 100. A high score shows good health according to the patient’s perspective. In subgroup analysis, score of patients with diabetes and without diabetes was compared.

The medical records of the patients, who were eligible for the quality of life assessment study, were reviewed. The preoperative demographics such as age, gender, accompanying diseases, perioperative and postoperative characteristics, and follow-up data were analyzed. The follow-up visits included physical examination, ankle-brachial index measurements and color-flow duplex scan if there was a decline at the ABI value. Patients, who had significant stenosis or abnormal flow pattern, underwent digital subtraction angiography, computerized angiography or magnetic resonance angiography. All patients were on best medical treatment, which included risk factors modification, dual antiplatelet therapy and lipid-lowering treatment.

All data were analyzed on SPSS Statistical Software. Mann-Whitney U-test was used to compare subgroups of the cohort. Comparison of the subgroups was carried out by Pearson’s chi-square and Fischer’s exact test. A p-value <0.05 was considered to be statistically.

## RESULTS

**Fig.1:**
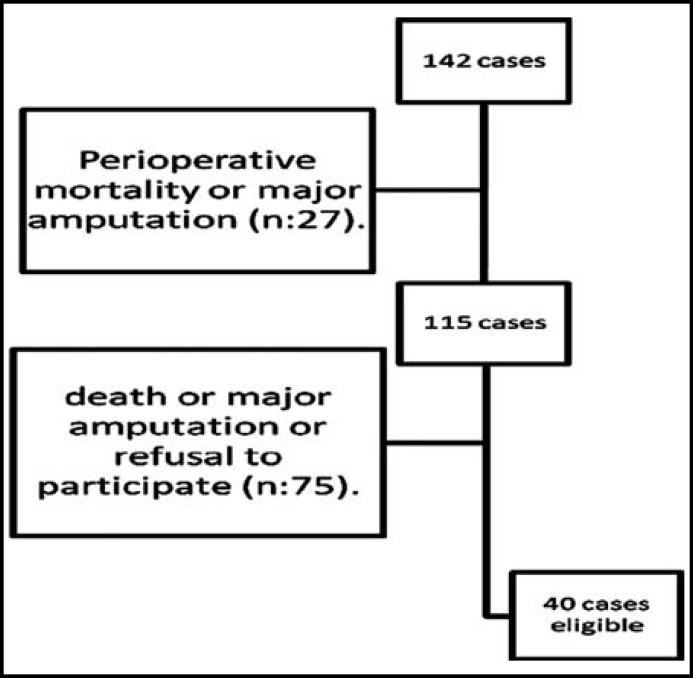
The inclusion rates to the study

There were 142 patients, who underwent a crural bypass for critical limb ischemia from 1999 to 2009 at our department. Twenty-seven (19%) patients died or lost their limbs during the 30-day perioperative period. There were 75 (52.8%) patients, who died or lost their ipsilateral or contralateral limbs during the follow-up or, who refused to participate or lost to follow-up. These patients were excluded from the study. There were 40 (28.2%) patients left available for the QoL assessment (Fig.1). The mean follow-up was 58±8 months at the assessment. 

Retrospective analysis of the 40 patients, who were included in the study, showed that 33 (82.5%) patients were male and 7 (17.5%) patients were female. The mean age at the time of intervention was 57±14 (33-83) years. Eighteen (45%) of the patients were diabetic, whilst 22 (55%) were non-diabetic. The frequency of Buerger Disease in this cohort was 35% (14/40). Hypertension, hyperlipidemia, ischemic heart disease, and end-stage renal disease were encountered in 24 (60%) patients, 11 (27.5%) patients, 19 (47.5%) patients, and 2 (5%) patients, respectively (Table-I).

All patients’ underwent digital subtraction angiography for evaluation of vascular disease. A percutaneous transluminal balloon angioplasty at the proximal site was carried out in 9 (22.5%) patients before surgery. The procedures were performed under general anesthesia in 23 (57.5%) patients whilst epidural anesthesia was used in 17 (42.5%) patients. The inflow artery was common femoral artery, superficial femoral artery, popliteal artery and posterior tibial artery in 7 (17.5%), 15 (37,5%), 17 (42.5%) patients and 1 (2.5%) patient, respectively. The outflow artery was posterior tibial artery, peroneal artery, dorsalis pedis artery and anterior tibialis artery in 16 (40%), 10 (25%), 6 (15%) patients and 8 (7.5%) patients, respectively. An autologous vein was used in 36 (90%) patients as a conduit, whilst a polytetraflouroethylene graft was used in 4 (10%) patients. Along with the bypass procedures, a toe amputation was carried out in 27 (65%) patients, whilst a transmetatarsal amputation was carried out in 13 (32.5%) patients. The mean duration of hospital stay was 26±6 (6-122) and 22±5 (8-100) days for the diabetic and non-diabetic population, respectively. The demographics in diabetic and non-diabetic population is shown on Table-II.

During the 30-days perioperative period, 3 (7.5%) patients suffered a superficial surgical site infection and 1 (2.5%) suffered a deep surgical site infection. Three (7.5%) patients suffered from graft thrombosis and the grafts were salvaged with thrombectomy. These patients did not require any further intervention.

**Fig.2:**
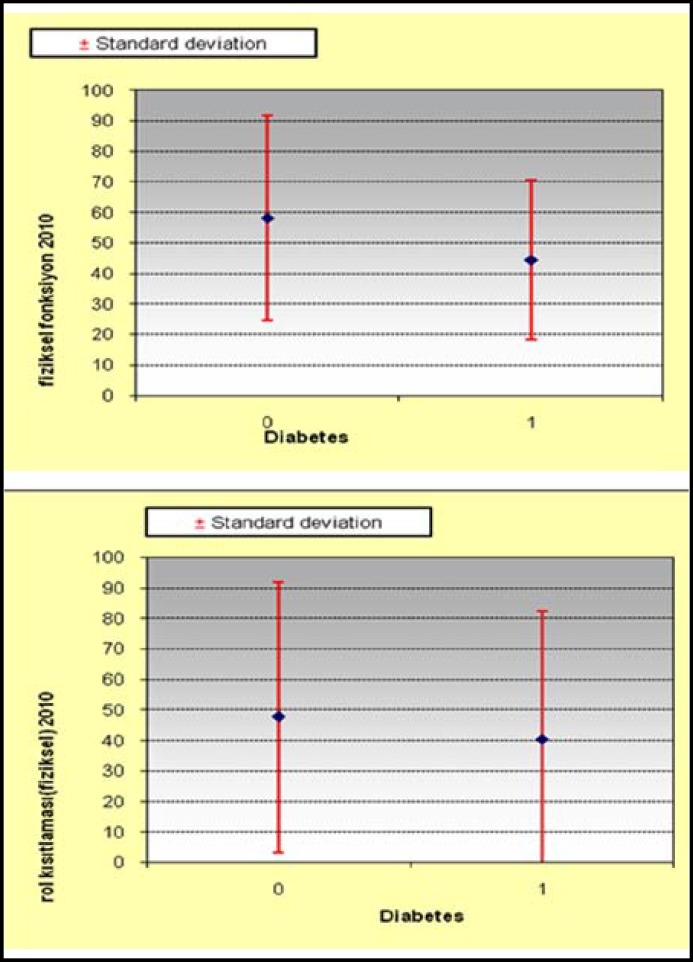
The comparison of physical functioning and role physical between patients with diabetes and without diabetes. p>0.05

The mean scores of the total group were 53,07±18.55 (10-87) in general perception of health, 52±30.85 (0-100) in physical functioning, 44.37±42.92 (0-100) in role-physical, 51.52±43.32 (0-100) in role-emotional, 67.93±21.14 (12.5-100) in social functioning, 63.65±25.16 (22-100) in bodily pain, 61.7±17.95 (12-92) in mental health, and 56.37±17.75 (20-90) in vitality (Fig.2, Table-III). The mean scores in the diabetic group were 48.94±18.86 (10-72) in general perception of health, 44.44±26.28 (0-100) in physical functioning, 40.28±42.13 (0-100) in role-physical, 51.72±44.61 (0-100) in role-emotional, 68.75±22.38 (25-100) in social functioning, 63.94±24.93 (22-100) in bodily pain, 59.11±20.14 (12-88) in mental health, and 53.89±16.76 (20-90) in vitality (Table-IV). The mean scores in the non-diabetic group were 56.45±18.2 (25-87) in general perception of health, 58.18±33.47 (10-100) in physical functioning, 47.73±44.26 (0-100) in role-physical, 51.36±43.31 (0-100) in role-emotional, 67.27±20.59 (12.5-100) in social functioning, 63.41±25.94 (22-100) in bodily pain, 63.82±16.12 (32-92) in mental health, and 58.41±18.67 (20-90) in vitality. There was no statistical difference concerning the mean scores of any dimensions of SF-36 questionnaire between the diabetic and non-diabetic population.

## DISCUSSION

Infrageniculate bypass is effective in most of the cases in preventing a major amputation.^2^ However, not every limb that is salvaged is associated with a good functional outcome and a better quality of life. Our results show that although not excellent, limbs that are salvaged with crural or pedal bypasses may provide a moderate quality of life and limb function despite an associated minor amputation. We have also seen that once the limb is salvaged, there is no difference between diabetic and non-diabetic population in the long term.

**Table-I:** Demographics of the patients, who were included in the study

	***Patients n (40)***		
*Demographics *	Mean Age (at intervention)		57 (33-83)
	Male		33 (82.5%)
*Risk Factors *	Diabetes Mellitus		18 (45%)
	Hypertension		24 (60%)
	Hyperlipidemia		11 (27.5%)
	Buerger’s Disease		14 (35%)
	CABG		6 (15%)
	Myocardial Infarction		16 (40%)
	Congestive Heart Failure		5 (12.5%)
	Transient Ischemic Attack		2 (5%)
	Stroke		9 (22.5%)
	End-stage Renal Insufficiency		2 (5%)
	Smoking	Never smoked	25 (62.5%)
		Ex-smoker	13(32.5%)
		Current smoker	2 (5%)

**Table-II:** Demographic of patients with and without diabetes

	***Diabetics N=18***		***Non-Diabetics N=22***		
***Demographics***	***N***	***%***	***N***	***%***	***p***
Male gender	14	77.7	19	86.3	0.25
Hypertension	14	77.7	10	45.4	0.03
Hyperlipidemia	6	33.3	5	22.7	0.45
IHD	12	66.6	7	31.8	0.02
COPD	6	33.3	6	27.2	0.67
ESRD	2	11.1	0	0	0.05
Smoking	6	33.3	9	40.9	0.62

IHD: Ischemic heart disease, COPD: Chronic Obstructive Pulmonary Disease,

ESRD: End-Stage Renal Disease, Smoking: Current smoker.

**Table-III:** Quality of Life measurements in the study population

***SF-36 score (Whole Group)***	***N***	***Minimum***	***Maximum***	***Mean***	***Std. Deviation***
General perception of health	40	10,00	87,00	53,07	18,55
Physical functioning	40	0,00	100,00	52,00	30,85
Role physical	40	0,00	100,00	44,37	42,92
Role emotional	40	0,00	100,00	51,52	43,32
Social functioning	40	12,50	100,00	67,93	21,14
Bodily pain	40	22,00	100,00	63,65	25,16
Mental health	40	12,00	92,00	61,70	17,95
Vitality	40	20,00	90,00	56,37	17,75

**Table-IV:** Comparison of eight domains of Short Form 36 between patients with and without diabetes

	***Non-Diabetic Patients***		***Diabetic Patients***	
	***Mean***	***Std Deviation***	***Mean***	***Std Deviation***
General perception of health	56,45	18,02	48,94	18,86
Physical functioning	58,18	33,47	44,44	26,28
Role physical	47,73	44,26	40,28	42,13
Role emotional	51,36	43,31	51,72	44,61
Social functioning	67,27	20,59	68,75	22,38
Bodily pain	63,41	25,94	63,94	24,93
Mental health	63,82	16,12	59,11	20,14
Vitality	58,41	18,67	53,89	16,76

The functional outcome and quality of life (QoL) assessments are well established tools for patient-oriented outcome measures. Short Form-36 is a validated generic questionnaire and functional outcome scales of this questionnaire may be used to assess if the limb covers the functional need of the patient or not.^4^ We decided to use SF36 test at this study because patients with peripheral arterial disease have accompanying diseases such as hypertension, ischemic heart disease and stroke, which may deteriorate the general status of the patient. Therefore, SF-36 is a generic test that covers the need of an overall QoL assessment in PAD patients. 

Studies have revealed that QoL is negatively affected by peripheral arterial disease.^5^^,^^6^ Moreover, it is shown that detrimental effect of the disease on QoL is worse in patients with critical limb ischemia when compared to patients with claudication.^7^ Recently published BASIL Trial, which is a prospective randomized multicenter trial, compares the efficacy and safety of open surgery and percutaneous balloon angioplasty in critical limb ischemia.^8^ However, the secondary outcomes of the trial include the assessment of QoL with SF-36 questionnaire. BASIL Trial results show that SF-36 scores at the follow-up are higher when compared to baseline scores. These positive effects are mainly evident in the dimensions of physical and mental scores. However, they are reflected the same in the long term period. Although this is a well designed prospective trial, in terms of QoL assessments, it is criticized that the patient population is not homogenous. The trial included more than 500 patients but not all patients underwent the same method of treatment. Moreover, the effect of accompanying diseases on QoL is not discussed in the paper.

The largest study to evaluate the effect of critical limb ischemia on QoL is PREVENT III Study.^9^ PREVENT III was a large, multicenter, double-blind, randomized clinical trial evaluating the efficacy of intraoperative treatment of vein grafts with edifoligide to prevent vein graft failure in patients undergoing infrainguinal arterial reconstruction with vein grafts for CLI. As a part of PREVENT III, the effect of infrainguinal vein grafting for limb salvage in CLI patients on health-related QoL was prospectively assessed by using the Vascular Quality of Life Questionnaire (VascuQol), which is a validated, standardized questionnaire. Patients were assessed preoperatively and at 3, 12 months postoperatively. The authors conclude that patients with CLI have a low QoL at baseline that is improved after lower extremity vein bypass and successful revascularization can be expected to improve QoL in patients with CLI, with benefits that are sustained to at least one year. The mean follow-up of the patients in our study was 58±8 (1-121) months. We are aware that non-homogenous follow-up period is a flaw of the study. We tried to overcome this problem by using means and standard deviations. The lack of a preoperative QoL assessment may well be regarded as a second limitation of the study. However, all previous papers in common already report that postoperative QoL is improved after infrainguinal bypass for critical limb ischemia.^2^ While conducting this study, the main idea was to evaluate the functional outcome of the limbs salvaged despite a minor amputation in the long term. Our initial evaluation revealed that all scales of the SF-36 questionnaire ranged from 50 to 60% in most of the patients. 

Some authors report that improvement in QoL after infrainguinal bypass is slower in diabetic patients and QoL scores are lower in this cohort.^4^ In a prospective study, it was revealed that improvement of QoL in non-diabetic patients were completed earlier when compared to diabetic patients and maximum improvement of quality of life was delayed in diabetics and less pronounced. In this study, which included 47 patients the authors conclude that QoL remained inferior in diabetics compared to non-diabetics throughout follow-up except for baseline-values, and they add that although non-diabetic patients maintained improvement in 5 domains, diabetic patients maintained improvement only in bodily pain at the end of the study, which lasted for 24 months. In our series, the mean scores of diabetic population were inferior to the mean scores of non-diabetic population in all dimensions. However, they were not statistically significant. Therefore, we have not encountered any scientific difference between the diabetic and non-diabetic patient population in order to conclude that we agree with the previous findings. We should also emphasize that the frequency of hypertension and ischemic heart disease was significantly higher in the diabetic group. This may at least explain why general perception of health and functional outcome measures were worse in diabetics. 

We have to be careful when analyzing the results of SF 36 questionnaire because every person feels different regarding the diseases and not all patients have common expectations from life. A patient may feel satisfied only with a neighbor visit whilst another will look for a total satisfaction from her/his business. Therefore, physical domain in SF-36 scales might be more informative. It is not always possible to salvage the foot totally. In certain cases only the heel may be salvaged. In our series, all patients had undergone a minor amputation ranging from toe amputation to transmetatarsal amputation. This is a special feature of this paper that is unique. We have not encountered any significant difference in terms of functional outcome scores in our patients with different levels of amputation. Therefore, it seems that salvage of the heel is sufficient to keep these patients ambulatory and maintain a satisfactory outcome. In another study of functional assessment, which included mostly diabetic patients revealed that more than 80% of patients were ambulatory following a transmetatarsal amputation.^10^^,^^11^ In accordance with this study, another one has shown that once the transmetatarsal stump is healed patients may return to their previous daily activities.^11^ It should also be noted that better results can be achieved in the young population.^12^

## CONCLUSION

In conclusion, infrageniculate bypasses are justified to avoid major amputations and help to maintain the independence of most of the patients. Although they may have complications with a minor amputation, the functional outcome is favorable and diabetes does not seem to have detrimental effect on the functional outcome and QoL. However, further studies with a larger sample size are needed to confirm our findings.

## Notes

***Disclosures:*** Kivanc Derya PEKER and Co-author do not have conflict of interest.
